# Allograft reconstruction of distal biceps ruptures: surgical technique and analysis of outcomes in chronic tears and failed repairs

**DOI:** 10.1016/j.xrrt.2025.100661

**Published:** 2026-01-02

**Authors:** Sameer R. Khawaja, Victoria A. Conn, Brianna L. Siracuse, Krishna N. Chopra, Amalie Erwood, Eric R. Wagner, Spero G. Karas

**Affiliations:** a Department of Orthopaedic Surgary, Baylor College of Medicine, Houston, TX, USA; b Department of Orthopaedic Surgery, Emory University School of Medicine, Atlanta, GA, USA

**Keywords:** Distal biceps ruptures, Chronic distal biceps tears, Failed biceps repair, Biceps rerupture, Allograft reconstruction, Distal biceps repair

## Abstract

**Background:**

Distal biceps tendon tears are relatively uncommon injuries that may result in pain and dysfunction. When these injuries are addressed acutely, healing rates, functional recovery, and patient outcomes are excellent. Despite generally good outcomes, failures do occur. Chronic tears of the distal biceps or those requiring revision surgery pose a unique challenge, as the tendons are often retracted and atrophic, requiring allograft reconstruction to bridge the gap in the repair construct. This manuscript aims to describe a safe, reproducible surgical technique and examine the clinical outcomes of distal biceps tendon reconstructions performed with allografts.

**Methods:**

A retrospective review of patient records from 2000 to 2022 to include Current Procedural Terminology code 24342 for reinsertion of biceps tendon, distal, with or without tendon graft, was performed. Operative notes were screened to determine which procedures included a distal biceps repair with an allograft. Charts were reviewed to determine a patient's most recent documented range of motion, pain score, and any postoperative complications. Patients were contacted by phone to determine their Quick Disabilities of Arm, Shoulder and Hand, Mayo Elbow Performance Score, and 12-Item Short Form Health Survey (SF-12) scores, as well as current visual analog scale for pain.

**Results:**

Fifteen patients who underwent distal biceps repairs with allograft were identified, and we were able to contact 10 (66%) of them. Six of these patients had chronic tears, while four had recurrent ruptures of previously performed acute distal biceps repairs. None of these patients reported complications or revision procedures. The average follow-up time for patients was 54 months (range: 8-182 months). The average visual analog scale pain score was 0.4 (range: 0-2). Average final flexion–extension was documented as 3°-130°, and prono–supination as 79°-89°. Patients reported an average Mayo Elbow Performance Score of 93.5 (range: 80-100), Quick Disabilities of Arm, Shoulder and Hand score of 4.8 (range: 0-15.9), SF-12 physical score of 54.3 (range: 50-57), and SF-12 mental score of 57.6 (range: 51-62).

**Conclusion:**

Allograft reconstruction of distal biceps injuries for chronic and recurrent tears results in excellent patient-reported outcomes and range of motion, with no significant complications among our cohort. Future studies with larger patient populations will help further validate our results.

Distal biceps tendon ruptures are uncommon injuries with an approximate incidence of 2.55 per 100,000 patient-years.^18^ The majority of patients who sustain these injuries are middle-aged men.^27^ The most common mechanism of injury is excessive eccentric load on a flexed elbow during strenuous activities such as weightlifting or labor-intensive jobs.^27^ These ruptures are due to the tendon having a vascular watershed near its insertion on the radial tuberosity.^1^^,^^29^ While proximal biceps tendon ruptures can be treated conservatively, most distal injuries require surgical repair.^24^^,^^32^ Distal biceps tendon repairs can be performed through a single- or double-incision approach, with both providing similarly successful results in acute settings.^10^ Common complications of distal biceps tendon repairs, such as transient nerve injury or heterotopic ossification, can occur in up to 25% of cases.^4^^,^^6^^,^^8^^,^^23^ Retears after surgery are uncommon, with reported recurrence rates of 1.6%-4%.^4^^,^^10^, ^11^, ^12^ These patients may require revision surgery to repair or reconstruct the ruptured tendon.

Chronic distal biceps tears, which are tears that occurred more than three weeks prior to surgical intervention, are also uncommon but are plagued with higher rates of postoperative complications.^3^^,^^15^ Prior studies have shown a preference for primary repair of these chronic ruptures over the use of a graft for reconstruction, even if it requires repairing the tendon in extreme elbow flexion.^3^ However, primary repair of chronic distal biceps tendon tears is not always possible, and reconstruction with an allograft tendon is required.^9^^,^^20^

Distal biceps tendon reruptures and chronic tears pose a unique challenge, as the tendons are often atrophied, retracted, and fibrosed, which can make it extremely difficult to repair the tendon directly.^20^ These cases often require allograft or autograft reconstruction or augmentation, with potential graft options including autograft fascia lata and semitendinosus tendons or allograft tibialis or Achilles tendons.^7^^,^^9^^,^^13^^,^^21^ Indications for a distal biceps repair with allograft augmentation depend on the setting of injury. For chronic tears, muscle atrophy and shortening, retraction of the distal tendon, and fibrosis are all common indications for allograft reconstruction vs. a primary anatomic reattachment.^9^ With chronic partial or complete tears, propagation of tears proximally into the musculotendinous junction can leave little to no healthy tendon for suture fixation. In these cases, allograft augmentation or reconstruction can allow for restoration of tendon length without the tendency for flexion contractures.^9^^,^^19^ Possible contraindications for surgical repair include significant comorbidities, active infection, restrictions in passive elbow and forearm motion, low functional demands, and a compromised soft tissue envelope.^32^

Some studies have found that Achilles tendon allografts provide a sufficient graft option while lowering morbidity in the reconstruction setting.^14^ Primary repairs augmented with allografts have been shown to produce similar failure and reoperation rates, final range of motion (ROM), and patient-reported outcome measures (PROMs) to those without allografts.^16^ While current literature has examined outcomes of primary distal biceps reconstructions with allografts for chronic tears,^9^^,^^16^^,^^20^^,^^26^^,^^30^ very few studies examine the long-term outcomes of revision distal biceps tendon reconstructions with allografts to correct reruptures. To our knowledge, no study has examined allograft augmentation for surgically repaired biceps tendons that have reruptured.

It is important to consider long-term outcomes when treating patients with chronic and recurrent distal biceps tendon ruptures. This study aims to describe the surgical technique of distal biceps repair with allograft augmentation or reconstruction for chronic and recurrent tears and examine the long-term outcomes of patients who underwent this procedure. We hypothesize that the use of an allograft provides excellent clinical outcomes with few observed complications.

## Methods

Following institutional review board approval, we performed a retrospective review of patient records at our institution. One hundred eighty-eight patients were identified using Current Procedural Terminology code 24342 for reinsertion of the biceps tendon, distal, with or without tendon graft, between 2007 and 2022. All charts were screened to identify patients who underwent distal biceps repairs with allograft reconstruction or augmentation for chronic tears or revision procedures for reruptures. Patients who underwent a primary acute repair were excluded from the study.

### Surgical technique

After regional anesthesia is performed in the preoperative holding area, the patient is taken to the operating room, where general anesthetic is induced. Patients are prepared and draped to the axilla in a standard sterile fashion. Tourniquets are not necessary but can be applied sterilely to the upper brachium if necessary. A 3-cm transverse incision is made at the upper antebrachium approximately 7 cm distal to the antecubital fossa. Care is taken to identify and protect the lateral antebrachial cutaneous nerve. In cases of revision surgery, the previous incision can be utilized where appropriate. Through the distal incision, the plane between the extensor–supinator mass laterally and the pronator teres and wrist flexors medially are developed. A blunt levering retractor is placed medially, and a right-angle retractor is placed laterally to avoid compression of the posterior interosseous nerve. Dissection down to the radial tuberosity then ensues, followed by débridement of soft tissue and/or any remnants of previous surgery. The bone is prepared with a curette. Two 3.5-mm pilot holes are drilled, and two 4.5-mm threaded anchors (Arthrex, Naples, FL, USA) are inserted.

**Figure 1:**
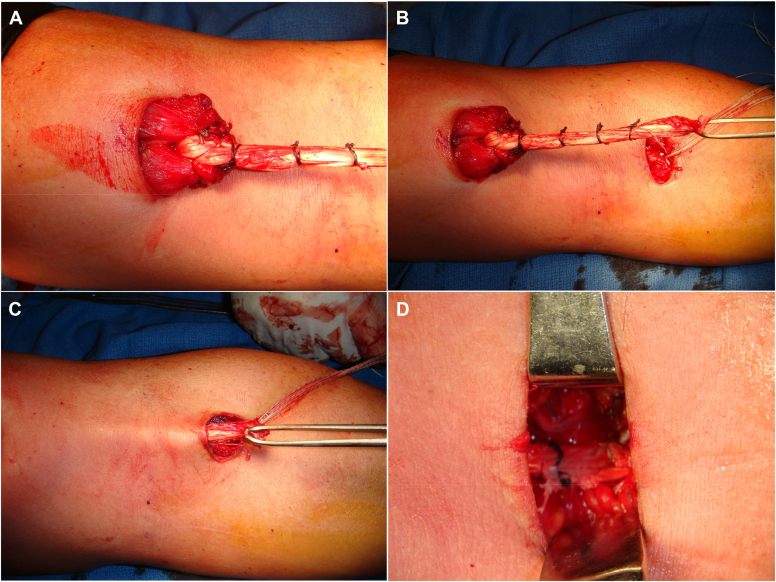
Graft insertion technique. (**A**) Distal biceps stump tied to allograft; (**B**) allograft length confirmed relative to insertion at radial tuberosity; (**C**) allograft tunneled to distal incision; and (**D**) allograft secured distally at radial tuberosity.

Attention is now turned proximally. A second transverse incision is made over the distal biceps muscle belly, and the distal biceps is presented through this incision. The distal biceps tendon is then débrided and prepared for the graft. Grafting can be performed with one of two methods: (1) fixation proximally to the distal biceps followed by attachment to the radial tuberosity (Fig. 1, *A*), or (2) the graft may be fixed to the radial tuberosity first and then tunneled subcutaneously to the proximal incision with subsequent fixation to the distal biceps. Regardless of technique, length–tension relationships must be recognized and established (Fig. 1, *B*). Grafts are fixed proximally with a Pulvertaft weave between the graft and distal biceps complex. The graft is fixed distally to the radial tuberosity with locking Krackow sutures and a tension-slide technique with a suture from each anchor (Fig. 1, *C*). This provides a “pulley” that seats the end of the allograft securely to bone. Each of the other sutures from each anchor are stitched in an interrupted fashion for secondary fixation of the distal construct (Fig. 1, *D*).

The wound is then thoroughly irrigated, and 500 mg of vancomycin powder is applied to each incision. The skin is closed in two layers. A sterile surgical dressing is applied, and a long-arm splint is placed.

### Postoperative protocol

Patients were instructed to follow a standard postoperative protocol. They remained in a long-arm splint for two weeks. At two weeks postop, the patient was fitted with an adjustable, hinged postop elbow brace (Ossur HF, Reykjavik, Iceland) set at 60°-90° of flexion. The flexion block was maintained at 90°, while the extension block was opened 10° per week over a 4-week period. After six weeks, the brace was discontinued, and a gentle ROM and strengthening program ensued and progressed until six months postoperatively, when most patients were discharged to full activity with only limited precautions.

### Data collection

Once patients of interest were identified, charts were reviewed for the following data: age, date of primary surgery (if applicable); date of revision surgery (in those patients having failed primary repair); intraoperative findings; surgical technique (single- vs. double-incision); fixation technique (suture anchor, intraosseous screw, cortical button, or bone tunnel); type of graft used; most recent ROM values; length of follow-up; complications; and any reoperations. A single-incision surgical technique was defined as a volar incision distal to the elbow flexion crease, while a double-incision surgical technique included a dorsal incision distal to the radiocapitellar joint.^2^ All patients were contacted by phone to complete functional outcome questionnaires to evaluate postoperative function and quality of life. PROMs collected included the Mayo Elbow Performance Score (MEPS), Quick Disabilities of Arm, Shoulder and Hand (QuickDASH), 12-Item Short Form Health Survey (SF-12) physical and mental scores, and current visual analog scale (VAS) pain scores.^12^^,^^22^^,^^23^^,^^25^

### Statistical analysis

Statistical analysis was performed on IBM SPSS Statistics (v. 29.0; IBM Corp., Armonk, NY, USA). Descriptive statistics were calculated to determine means and standard deviations of all PROMs within both the rerupture and chronic tear cohorts and overall. The Shapiro–Wilk test was used to determine normality of the data, during which all outcome data were found to be normally distributed except for VAS pain. Student *t*-tests were used to compare outcomes data between both groups for MEPS, QuickDASH, and SF-12 physical and mental measures. The Mann–Whitney *U* test was used to compare VAS pain between groups.

## Results

We identified 15 patients who underwent chronic or revision distal biceps repairs or reconstructions with allografts between 2007 and 2022. Of the 15, nine patients underwent primary surgery for a chronic biceps tendon tear, and six underwent revision surgery for a rerupture. Seven cases underwent fixation using suture anchors, six used cortical buttons, and the remaining cases were managed with varying techniques. Eleven cases used a single-incision approach, and four used a double-incision approach.

All patients in the study were male, with an average age of 51 years (range: 31-67). The average postoperative follow-up was 54 months (range: 8-182). All patients demonstrated improved ROM following surgical intervention, with similar clinical findings between the rerupture and chronic tear cohorts. At the most recent clinic visit, average flexion–extension was 2.9°-130.0°, and prono–supination was 79.4°-88.8°. When comparing the rerupture cohort to the chronic tear cohort, average final flexion–extension was 4.6°-130.0° vs. 0°-130.0°, and prono–supination was 82.0°-90.0° vs. 75.0°-87.7°, respectively. Fourteen of the 15 patients also had completely resolved pain by their final clinic visits, with the final patient rating his pain as 2/10. No patient experienced any complications with their procedure, nor did any patient return to the operating room for revision surgery.

Ten of the 15 patients could be reached by telephone to collect updated PROMs (Table I). Six of the patients underwent primary repair for a chronic tendon tear, and four underwent revision for a rerupture. Overall PROM values demonstrated an average VAS pain score of 0.4 (range: 0-2), MEPS of 93.5 (range: 80-100), QuickDASH of 4.8% (range: 0%-15.9%), and SF-12 physical and mental scores of 54.3 (range: 50-57) and 57.6 (range: 51-62), respectively. When comparing PROMs based on etiology of injury (Table II), the chronic tear cohort had a higher mean SF-12 mental score compared to the rerupture cohort, 60.4 ± 1.4 vs. 53.4 ± 3.8 (*P* = .003), respectively. No significant differences were found when comparing PROMs between the single- and double-incision cohorts.

**Table I:** Fixation type, graft type, and patient-reported outcomes data following distal biceps reconstruction with allograft.

Patient	Etiology	Graft type	Fixation technique	VAS pain	Mayo Elbow Performance Score	QuickDASH (%)	SF-12 (physical)	SF-12 (mental)
1	Rerupture	Palmaris longus	Suspensory fixation	1	80	9.1	52.0	59.7
2	Rerupture	Achilles	Suspensory fixation	1	100	9.1	53.1	62.6
3	Rerupture	Achilles	Suspensory fixation	0	100	0	56.6	60.8
4	Rerupture	Achilles	Suspensory fixation	0	100	0	56.8	57.9
5	Rerupture	Semitendinosus	Suspensory fixation	1	95	9.1	57.6	36.7
6	Rerupture	Achilles	Suspensory fixation	1	100	6.8	56.6	60.8
7	Chronic tear	Tibialis anterior	Suspensory fixation	5	100	4.5	55.3	60.7
8	Chronic tear	Tibialis anterior	Suture anchor	0	85	4.5	53.0	53.4
9	Chronic tear	Achilles	Suspensory fixation	0	95	4.5	56.0	51.1
10	Chronic tear	Achilles	Suture anchor	0	100	0	57.2	58.8
Mean ± SD				0.9 ± 1.5	95.5 ± 7.2	4.76 ± 3.8	55.4 ± 1.8	56.2 ± 7.7

*SD*, standard deviation; *VAS*, visual analog scale; *QuickDASH*, Quick Disabilities of Arm, Shoulder and Hand; *SF-12*, 12-Item Short Form Health Survey.

**Table II:** Comparison of postoperative patient-reported outcomes between reruptured biceps tendon and chronic tear cohorts.

	Rerupture (n = 6)	Chronic tear (n = 4)	*P* value
VAS pain, mean ± SD	0.7 ± 0.5	1.3 ± 2.5	.677
Mayo Elbow Performance Score, mean ± SD	95.8 ± 7.1	95 ± 7.1	.868
QuickDASH (%), mean ± SD	5.7 ± 4.5	3.4 ± 2.3	.315
SF-12 (physical), mean ± SD	55.5 ± 2.3	55.4 ± 1.8	.946
SF-12 (mental), mean ± SD	56.4 ± 9.8	56.0 ± 4.5	.930

*SD*, standard deviation; *VAS*, visual analog scale; *QuickDASH*, Quick Disabilities of Arm, Shoulder and Hand; *SF-12*, 12-Item Short Form Health Survey.

## Discussion

This study examined surgical outcomes of chronic and revision distal biceps procedures. Distal biceps ruptures are an uncommon injury with an estimated incidence of 2.55 per 100,000 patient-years.^18^ Reruptures after surgical repair are also an uncommon phenomenon, with an incidence of approximately 1.5% after primary repair.^17^^,^^18^ While current literature has examined long-term outcomes of distal biceps repairs and reconstructions with an allograft to treat chronic ruptures,^16^ there remains a gap in the literature on outcomes for reruptured biceps tendons treated with allograft reconstruction or augmentation.

Previous studies have provided background to the broad topic of distal biceps tendon repairs. Hendy et al^16^ described the positive outcomes of using allograft augmentation to treat chronic distal biceps tears, finding similar failure rates, reoperation rates, ROM, and PROMs between allograft and nonallograft cohorts. Other studies have also found improved outcomes and limited complications for treating chronic tears with and without allografts.^12^^,^^16^^,^^28^ One study, however, found that chronic repairs without grafts have been shown to have deficits in flexion and supination strength and postoperative flexion contractures.^31^ Prokuski et al^23^ examined outcomes of revision distal biceps tendons without allografts and found overall postoperative improvements in DASH scores and the American Shoulder and Elbow Society - Elbow score self-assessment examinations; however, they also found a possible association with complications such as scarring and transient irritation of cutaneous nerves. Disadvantages to allograft augmentation, such as increased cost, the small risk of disease transmission,^26^ and limited availability, are offset by similar, if not improved outcomes,^30^ reduced complications, and alleviating concerns regarding size mismatch.^9^ Several other studies have also found similar results when comparing single- vs. double-incision repairs, confirming the results from our study.^5^^,^^11^^,^^12^

This study is the first to examine the use of allograft to treat both chronic and reruptured distal biceps tendons. While other studies have examined the use of allografts,^7^^,^^12^^,^^16^ they have focused on their application for chronic tears. Our findings demonstrate excellent outcomes of distal biceps tendon repairs and reconstructions with allografts for revisions secondary to reruptures and primary procedures for chronic tears. Our results also provide long-term outcomes for rerupture revisions of the distal biceps, demonstrating favorable outcomes and results that are similar to those for primary repairs to treat chronic tears. The use of allograft is a viable solution when anatomic repairs cannot be achieved with the native tendon by providing increased tendon length and avoiding flexion contractures.^9^ This study also examined single- vs. double-incision techniques, finding no notable differences between the two. These findings suggest the use of allografts as augments for repairs or grafts for reconstructions of the distal biceps in the setting of chronic or recurrent tears provide acceptable clinical outcomes without any observed complications.

Limitations of this study include its small sample size, lack of diversity within the sample, a large dependence on subjective outcomes, and incomplete follow-up. While 15 patients were included in the study with final, objective outcomes, only 10 could be contacted by telephone for updated PROMs. Therefore, we cannot determine whether the remaining five patients had as positive subjective outcomes as their objective clinical findings indicate. In addition, we do not have preoperative PROMs to compare to the postoperative ones. While no complications or further revisions were reported, and patients reported excellent outcomes at their final postoperative visit, this was a single-institution study, and we were unable to determine if the five remaining patients followed up at other institutions for any late-stage complications that required further surgery.

## Conclusion

Revision and chronic distal biceps tears occur infrequently, though repair techniques pose a unique challenge for orthopedic surgeons. This study presents a small cohort of patients who have undergone a repair or reconstruction of the distal biceps tendon using allografts with a safe and reproducible technique. All patients demonstrated excellent ROM at final follow-up with no reported complications. Patients also reported excellent outcomes several years following their procedures. Future studies should aim for larger and more diverse sample sizes to validate the results of our study.

## Disclaimers:

Funding: No funding was disclosed by the authors.

Conflicts of interest: Spero G. Karas, MD is a consultant and receives royalties from Smith and Nephew. He is also a consultant for Annika Therapeutics. No research funding from any company was used for or relevant to this study. The other authors, their immediate families, and any research foundation with which they are affiliated have not received any financial payments or other benefits from any commercial entity related to the subject of this article.
